# Chaihu-Shugan-San (Shihosogansan) alleviates restraint stress-generated anxiety and depression in mice by regulating NF-κB-mediated BDNF expression through the modulation of gut microbiota

**DOI:** 10.1186/s13020-021-00492-5

**Published:** 2021-08-14

**Authors:** Sang-Kap Han, Jeon-Kyung Kim, Hee-Seo Park, Yeun-Jeong Shin, Dong-Hyun Kim

**Affiliations:** Neurobiota Research Center and Department of Life and Nanopharmaceutical Sciences, College of Pharmacy, Kyung Hee University, 26, Kyungheedae-ro, Dongdaemun-gu, Seoul, 02447 Korea

**Keywords:** Chaihu-Shugan-San, Depression, Fecal microbiota transplantation, Gut microbiota

## Abstract

**Background:**

Chaihu-Shugan-San (CSS, named Shihosogansan in Korean), a Chinese traditional medicine, is frequently used to treat anxiety and depression. Psychiatric disorders including depression are associated with gut dysbiosis. Therefore, to comprehend gut microbiota-involved anti-depressive effect of CSS, we examined its effect on restraint stress (RS)-induced depression and gut dysbiosis in mice

**Methods:**

CSS was extracted with water in boiling water bath and freeze-dried. Anxiety and depression was induced in C57BL/6 mice by exposure to RS. Anxiety- and depression-like behaviors were measured in the light/dark transition and elevated plus maze tasks, forced swimming test, and tail suspension test. Biomarkers were assayed by using the enzyme-linked immunosorbent assay and immunoblotting. The gut microbiota composition was analyzed by Illumina iSeq sequencer.

**Results:**

CSS significantly reduced the RS-induced anxiety- and depression-like behaviors in mice. CSS suppressed the RS-induced activation of NF-κB and expression of interleukin (IL)-6 and increased the RS-suppressed expression of brain-derived neurotrophic factor (BDNF). Furthermore, CSS suppressed the RS-induced IL-6 and corticosterone level in the blood and IL-6 expression and myeloperoxidase activity in the colon. CSS decreased the RS-induced γ-Proteobacteria population in gut microbiota, while the RS-suppressed Lactobacillaceae, Prevotellaceae, and AC160630_f populations increased. Fecal transplantation of vehicle-treated control or RS/CSS-treated mice into RS-exposed mice significantly mitigated RS-induced anxity- and depression-like behaviors, suppressed the NF-κB activation in the hippocampus and colon, and reduced the IL-6 and corticosterone levels in the blood. These fecal microbiota transplantations suppressed RS-induced Desulfovibrionaceae and γ-Proteobacteria populations and increased RS-suppressed Lactobacillaceae and Prevotellaceae poulation in the gut microbiota.

**Conclusions:**

CSS alleviated anxiety and depression by inducing NF-κB-involved BDNF expression through the regulation of gut inflammation and microbiota.

**Supplementary Information:**

The online version contains supplementary material available at 10.1186/s13020-021-00492-5.

## Background

Anxiety is one of mental disorders characterized by chronic, exaggerated anxiety, worry, tension, and fear [1]. Patients with anxiety disorders generally progress to the depression [2]. Therefore, anxiety and depression share a lot of common symptoms. Exposure to stressors such as restraint stress and social defeat induces the release of glucocorticoids and adrenaline in the adrenal gland and interleukin (IL)-6 and tumor necrosis factor (TNF)-α in immune cells through the hypothalamo-pituitary-adrenal (HPA) axis [3–5]. These cytokines cause sickness and immobile behavior, which is very similar to the behavioral symptoms of depression in humans [6, 7]. The excessive secretion of glucocorticoids and inflammatory cytokines suppresses the expression of brain-derived neurotrophic factor (BDNF), which is closely associated with the occurrence of psychiatric disorders including depression through the modulation of neuroplasticity in the brain [8], in the hippocampus and activates the innate and adaptive immune responses in the gastrointestinal tract, resulting in the outbreak of anxiety, depression, gut inflammation, and gut dysbiosis [9–11]. Gut microbiota and their byproducts such as butyric acid and lipopolysaccharide regulate the enteric nervous and immune systems in the gastrointestinal tract, which modulate neurogenesis and neuroinflammation in the brain through the neuronal, neuroendocrine and neuroimmune routes [9, 12]. Gut dysbiosis is closely connected with the outbreak of systemic diseases such as psychiatric disorder and metabolic syndrome through microbiota-gut-brain (MGB) axis [13–15].

Chaihu-Shugan-San (CSS, named Shihosogansan in Korean), consists of Radix Paeoniae Alba, Radix Bupleuri, Fructus Aurantii, Pericarpium Citri Reticulatae, Radix Glycyrrhizae, Rhizoma Chuanxiong, and Rhizoma Cyperi, is a well-known traditional Chinese medicine formula for the therapy of anxiety and depression [16, 17]. CSS alleviates chronic stress-induced depression in rodents [18, 19]. CSS also mitigates the liver function and chronic metabolic inflammation in rats with nonalcoholic fatty liver disease [19]. Furthermore, it suppresses high-fat diet-induced the inflammatory response including proinflammatory cytokine expression and gut dysbiosis including increased Enterobacteriaceae and Staphylococcaceae population in rats [19]. Recently, Yu et al. reported that CSS alleviated imipenem/cilastatin/stress-induced depression and increased imipenem/cilastatin/stress-suppressed Proteobacteria and Firmicutes populations in mice [20]. Jang et al. reported that the disruption of gut microbiota, particularly increased Proteobacteria population, by antibiotic ampicillin significantly caused psychiatric disorder in mice by suppressing NF-κB mediated BDNF expression [21]. Gut microbiota-mediated anti-depressive mechanism of CSS remains unclear.

Therefore, we examined the gut microbiota-involved mechanism of CSS on restraint stress (RS)-induced anxiety and depression in mice.

## Methods

### Materials

An enzyme-linked immunosorbent assay (ELISA) kit for corticosterone was obtained from Elabscience (Hebei, China). ELISA kits for IL-6 and TNF-α were obtained from R&D systems (Minneapolis, MN). Antibodies for BDNF, p-p65, p65, and β-actin were obtained from Cell Signaling Technology (Beverly, MA). A QIAamp DNA stool mini kit was obtained from Qiagen (Hilden, Germany).Buspirone (PC) was obtained from Sigma-Aldrich (St Louis, MO).

### Preparation of CSS water extraction

All herbal medicines were purchased from Kyung Hee University, Korean Medical Center, Division of Herbal Medicine (Seoul, Korea) and authenticated by adjunctive Professor Nam-Jae Kim, Department of Oriental Medicine, Kyung Hee University. Voucher specimens (No. NKH19-1 ~ 7) were deposited at College of Pharmacy, Kyung Hee University (Seoul, Korea) and the formula of CSS prescription was prepared in accordance with The People׳s Republic of China Pharmacopoeia 1st volume conventional dosage (Table 1) [14]. CSS (65 g) was extracted with distilled water (1:12, w/v) in boiling water for 30 min twice. These supernatants were combined and freeze-dried (yield = 20.5%). Its HPLC chromatogram is indicated in Additional file 1: Figure S1.

**Table 1:** Ingredients of Chaihu-Shugan-San (CSS) formula

Herbal medicine	Amount (g)	number of voucher specimen
Radix Bupleuri (*Bupleurum chinese* DC.)	9.0	NKP19-1
Pericarpium Citri Reticulatae (*Citrus reticulate* Blanco)	9.0	NKP19-2
Rhizoma Chuanxiong (*Ligusticurn chuanxiong* Hort.)	9.0	NKP19-3
Rhizoma Cyperi (*Cyperus rotundus* L.)	9.0	NKP19-4
Fructus Aurantii (*Citrus aurantium* L.)	9.0	NKP19-5
Radix Paeoniae Alba (*Paeonia lactiflora* Pall.)	15.0	NKP19-6
Radix Glycyrrhizae (*Glycyrrhiza uralensis* Fisch)	5.0	NKP19-7

### Animals

C57BL/6 mice (male, 19–21 g, 6 weeks old) were supplied from Orient Bio (Seongnam-shi, Korea) and acclimated for 1 week and used in animal experiments. Mice were maintained in plastic cages with the raised wire floor (3 mice per cage) at the controlling condition (temperature; 20–22 ^○^C; humidity, 50 ± 10%; light/dark cycle, 12 h), fed with water and food ad libitum.

All animal experiments were approved by the Institutional Animal Care and Use Committee of the University (IACUC No KUASP(SE)-19-152) and carried out in accordance with the NIH and University Guide for Laboratory Animal Care and Usage.

### Preparation of mice with RS-induced anxiety and depression

An anxiety/depression mouse model was prepared by RS exposure in accordance with the method of Han et al. [22]. Briefly, to induce RS in mice, each mouse except normal control (NC) group was inserted into a 35-mL cylindrical instrument (7.5 cm in length and 2.5 cm in diameter) with a 0.25-cm-diameter hole on the tube center, fixed to restrict side-to-side and forward-to-backward mobility, and vertically installed for 12 h/day. RS was exposed once a day for 2 days. Each group contained six mice.

First, to decide the dose of CSS in mouse experiment, mice were randomly separated into five groups. Test agents (RS, vehicle (saline); CS_0.5_, 0.5 g/kg of CSS; CS_1.0_, 1 g/kg of CSS; CS_4.0_, 4 g/kg CSS, dissolved in saline) were orally (for vehicle and CSS) administered in RS-exposed mice daily for 5 days from 24 h after the final RS exposure. RS-untreated normal control mice (NC) were treated with saline (vehicle).

Second, mice were separated into four groups. Mice except NC were exposed to RS. Test agents (RS, vehicle; PC, 1 mg/kg buspirone; CS_1.0_, 1 g/kg of CSS) were orally gavaged (for CSS and saline) or intraperitoneally injected (for buspirone) in RS-exposed mice daily for 5 days from 24 h after the final RS exposure. NC was treated with saline.

Third, to understand the effect of fecal microbiota transplantation (FMT) on the occurrence of anxiety/depression, fecal microbiota suspension agents (NC, vehicle; RS, vehicle; FN, 0.2 mL of the fecal microbiota suspension of vehicle-treated mice; FR, 0.2 mL of the fecal microbiota suspension of RS-treated mice) were orally gavaged in (the stomach of) mice once a day for 5 days. NC were treated with saline (vehicle).

Fourth, to understand the effect of fecal microbiota transplantation (FMT) on the occurrence of anxiety/depression, fecal microbiota suspension agents (NC, vehicle; RS, vehicle; IFC, 0.2 mL of the fecal microbiota suspension of RS/CSS-treated mice [FC]; IFN, 0.2 mL of the fecal microbiota suspension of vehicle-treated mice [FN]) were orally gavaged in RS-exposed mice once a day for 5 days. NC were treated with saline (vehicle).

For the preparation of FC and FR, CSS and vehicle were orally treated daily for 5 day in RS-exposed mice, respectively. For the preparation of FN, vehicle were orally treated daily for 5 day in normal control mice. These feces (0.5 g) were collected 48 h after the final treatment with CSS or vehicle, suspended in saline on ice, centrifuged (500 g*,* 5 min, and 4 °C), washed with saline twice by centrifuging (10,000 g*,* 20 min, and 4 °C), and suspended in saline (4.5 mL).

Anxiety- and depression-like behaviors were monitored in the light/dark transition (LDT) and elevated plus maze (EPM) tasks, tail suspension test (TST), and forced swimming test (FST), 18 h after the final treatment with test agents. Mice were killed by CO_2_ inhalation. Bloods, brains, and colons were collected. Brain and colon tissues were stored at – 80 °C for the assay of biochemical markers.

### Behavioral tasks

Anxiety-like behaviors were evaluated in the EPM and LDT tasks and Depression-like behaviors were in the TST and FST. The EPM task was performed in the plus-maze apparatus (consisting of two open [30 × 7 cm] and two enclosed arms [30 × 7 cm] with 20-cm-high walls extending from a central platform [7 × 7 cm] on a single central support to a height of 60 cm above the floor) for 5 min, as reported previously [22]. The time spent in open arm (OT) and entry number into the open arm (OE) were counted. The LDT task was performed in the light/dark box apparatus (45 × 25 × 25 cm, consisting of two chambers made of black and white polywoods [walls] and Plexiglass [floor] connected by an opening [7.5 × 7.5 cm] located at floor level in the center of the dividing wall) for 5 min, as reported previously [11]. The time spent in the light box (TL) and number of transition into the light box entry (NT) were counted. The TST was performed for 6 min, as reported previously [23]. Mice were suspended on the edge of a table 30 cm above the floor by taping 1 cm from the tail tip. Immobility time was measured for 5 min. Mice were judged to be immobile, when they did not move and hanged passively. The FST was performed in a round transparent plastic jar (20 × 40 cm^3^) containing fresh water (25 °C) to a height of 25 cm, as reported previously [23]. Immobility time was measured during 5 min. Mice were judged to be immobile, when they remained floating in the water without struggling. Immobility (%) was calculated as immobility time/measured time × 100.

### Myeloperoxidase activity assay

Myeloperoxidase activity assay was performed, as previously described [22]. In the reaction mixture (1 mL) consisted of 0.03% hydrogen peroxide and 1.6 mM tetramethylbenzidine, the colon homogenate supernatant (50 μL) was added and the absorbance at 650 nm was successively monitored over 5 min. Activity was defined as the quantity degrading 1 μmol/mL of hydrogen peroxide.

To prepare the colon homogenate supernatant, mouse colons were homogenized with ice-cold radioimmunoprecipitation assay (RIPA) lysis buffer and centrifuged (10,000 g*,* 10 min, and 4 °C). The resulting supernatant was used as a crude enzyme supernatant.

### ELISA assay and immunobloting

For the assay of ELISA, hippocampus and colon tissues were homogenized with ice-cold RIPA lysis buffer consisted of and 1% protease inhibitor cocktail and 1% phosphatase inhibitor cocktail and centrifuged (10,000*g*, 10 min, and 4 °C) [24]. Bloods were collected from carotid artery and centrifuged (3000*g*, 5 min, and 4 °C). The resulting supernatants (50 μL) were transferred to 96-well plate. Cytokines and corticosterone levels were measured using ELISA kits according to the manufacturer’s protocol.

For the analysis of immunoblotting, the supernatants (20 ng of protein) were electrophoresed, transferred to a nylon membrane, incubated with the corresponding antibodies (p65 [1:1000], p-p65 (1:1000), BDNF [1:500], and β-action [1:1000]), washed with phosphate-buffered saline containing tween 20, incubated with horseradish peroxidase-conjugated secondary antibodies, and visualized with an enhanced chemiluminescence detection kit [23].

### Illumina iSeq sequencing

Gut bacterial 16S rRNA gene pyrosequencing was carried out, as reported previously [25]. First, the fresh feces were collected from mice and genomic DNA was extracted using QIAamp DNA stool mini kit. The genomic DNA was amplified using barcoded primers, which targeted the bacterial 16S rRNA gene V4 region. Pyrosequencing reads were deposited in the NCBI’s short read archive under accession number PRJNA649086.

### Statistical analysis

All data are expressed as the means ± standard deviation (SD) and conducted GraphPad Prism 8 (GraphPad Software, Inc., San Diego, CA). The significance was analyzed by Kruskal–Wallis test with Dunn's post-hoc test for non-parametric analysis (p < 0.05).

## Results

### CSS alleviated RS-induced anxiety and depression in mice

**Fig. 1:**
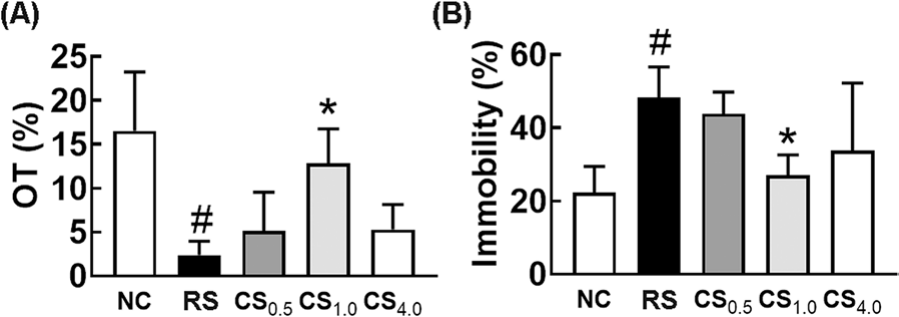
Effect of CSS on the restraint (immobilization) stress (RS)-induced anxiety and depression in mice. **A** Effect on RS-induced anxiety-like behaviors in the EPM (OT, time spent in the open arm). **B** Effect on RS-induced depression-like behaviors in the TST. Test agents (RS, vehicle [saline]; CS_0.5_, 0.5 g/kg of CSS; CS_1.0_, 1 g/kg of CSS; CS_4.0_, 4 g/kg CSS, dissolved in saline) was orally gavaged daily for 5 days from the next day after the final exposure to restraint stress (RS). Normal control mice (NC) was orally treated with vehicle (saline) instead of CSS. Data values were indicated as mean ± SD (n = 6). ^#^*p* < 0.05 *vs.* NC group. ^*^p < 0.05 *vs*. RS group

First, to decide the dosage of CSS in animal experiments, we investigated the effect of CSS on the RS-induced anxiety and depression in mice. Exposure to RS significantly decreased the time spent in the open arm (OT) in the EPM task and increased the immobility time in the TST. However, oral gavage of CSS dose-dependently suppressed the RS-induced anxiety- and depression-like behaviors: it increased RS-suppressed OT and suppressed RS-induced immobility (Fig. 1). Of these, CSS at a dose of 1.0 g/kg/day most potently alleviated depressive behaviors, as reported previously [17]. However, the high dose attenuated its effect.

**Fig. 2:**
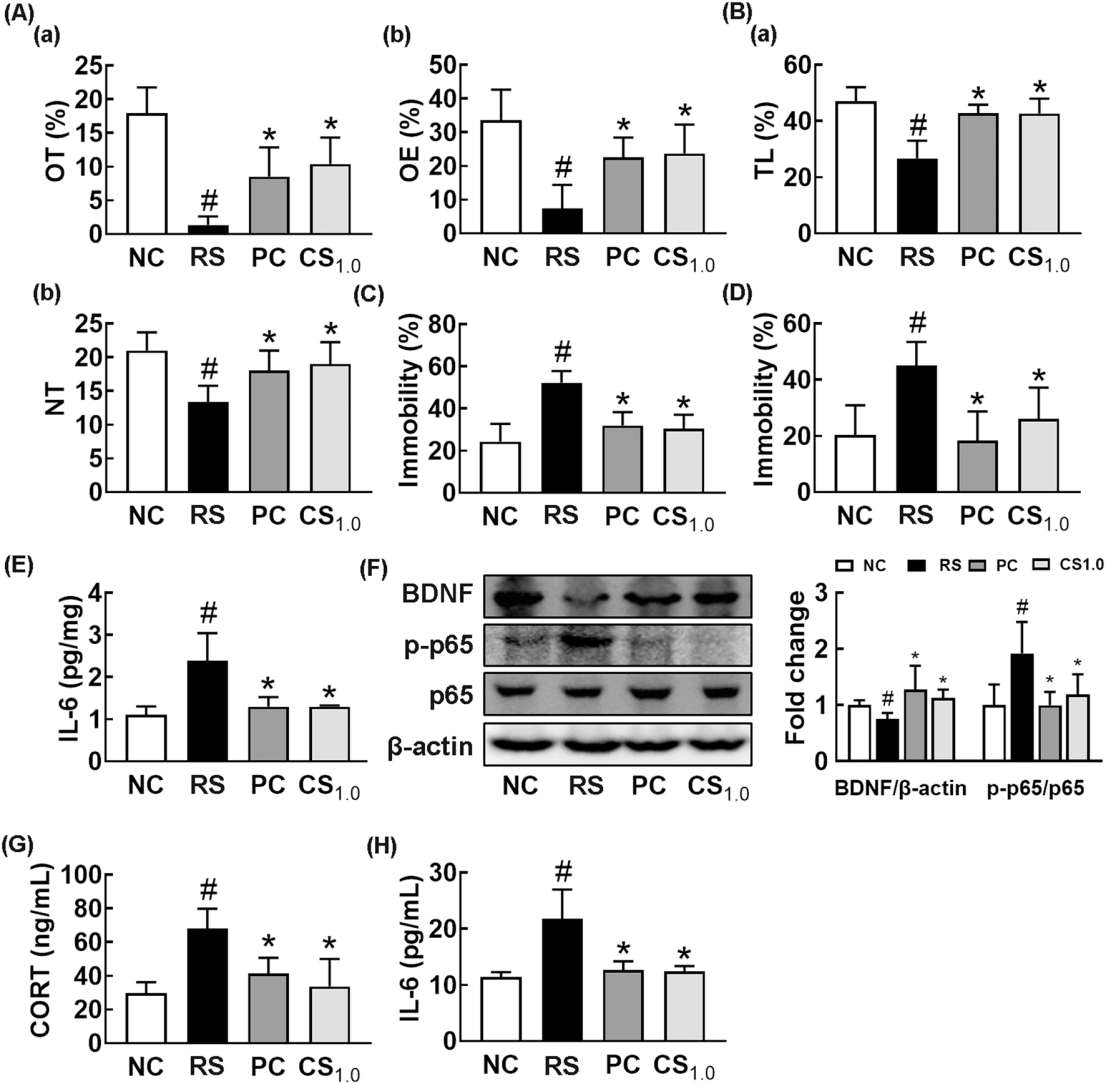
Orally gavage of CSS alleviated restraint stress (RS)-induced anxiety and /depression in mice. Effects on RS-induced anxiety/depression-like behaviors in the EPM (**A**: a, time spent in the open arm [OT]; b, open arm entries [OE]), LDT (**B**; a, time spent in the light box [TL]; b, number of transition into the light box entry [NT]), TST (**C**), and FST (**D**). **E** Effect on IL-6 (E) expression in the hippocampus, assessed by ELISA. **F** Effect on the BDNF expression and NF-κB activation (p-p65 to p65 ratio) in the hippocampus. Effect on corticosterone (CORT, G) and IL-6 levels (H) in the blood. Test agents (RS, vehicle [saline]; PC, (1 mg/kg/day of buspirone, i.p; CS_1.0_, 1 g/kg/day of CSS) were treated daily for 5 days from the next day after the final exposure to restraint stress (RS). Normal control mice (NC) was orally treated with vehicle (saline) instead of test agents. Data values were indicated as mean ± SD (n = 6). ^#^*p* < 0.05 *vs.* NC group. ^*^p < 0.05 *vs*. RS group

Therefore, to confirm the anti-depressive effect of CSS, we orally administered it at 1.0 g/kg/day in mice with RS-induced anxiety and depression and monitored anxiety- and depression-like behaviors. Exposure to RS significantly decreased the OT in the EPM task and TL in the LDT task to 7.5% and 56.8% of control group, respectively, and increased the immobility times in TST and FST to 215.6% and 221.9% of control group, respectively (Fig. 2A–D). However, CSS treatment increased the RS-suppressed OT to 58.1% of control group (Fig. 2A). Treatment with CSS increased the RS-suppressed TL in the LDT task to 70.3% of control group (Fig. 2B). CSS treatment also alleviated RS-induced immobility times in the TST and FST to 125.4% and 128.5% of control group, respectively (Fig. 2C, D). Exposure to RS decreased the expression of BDNF in the hippocampus, while the activation of NF-κB (p-p65 to p65 ratio) and expression of IL-6 increased (Fig. 2E, F). However, CSS significantly suppressed the RS-induced expression of IL-6 and activation of NF-κB (p-p65 to p65 ratio), while the RS-suppressed expression of BDNF increased. Oral administration of CSS also decreased the RS-induced IL-6 and corticosterone levels in the blood (Fig. 2G, H).

### CSS alleviated colitis and gut dysbiosis in mice with RS-induced anxiety and depression

**Fig. 3:**
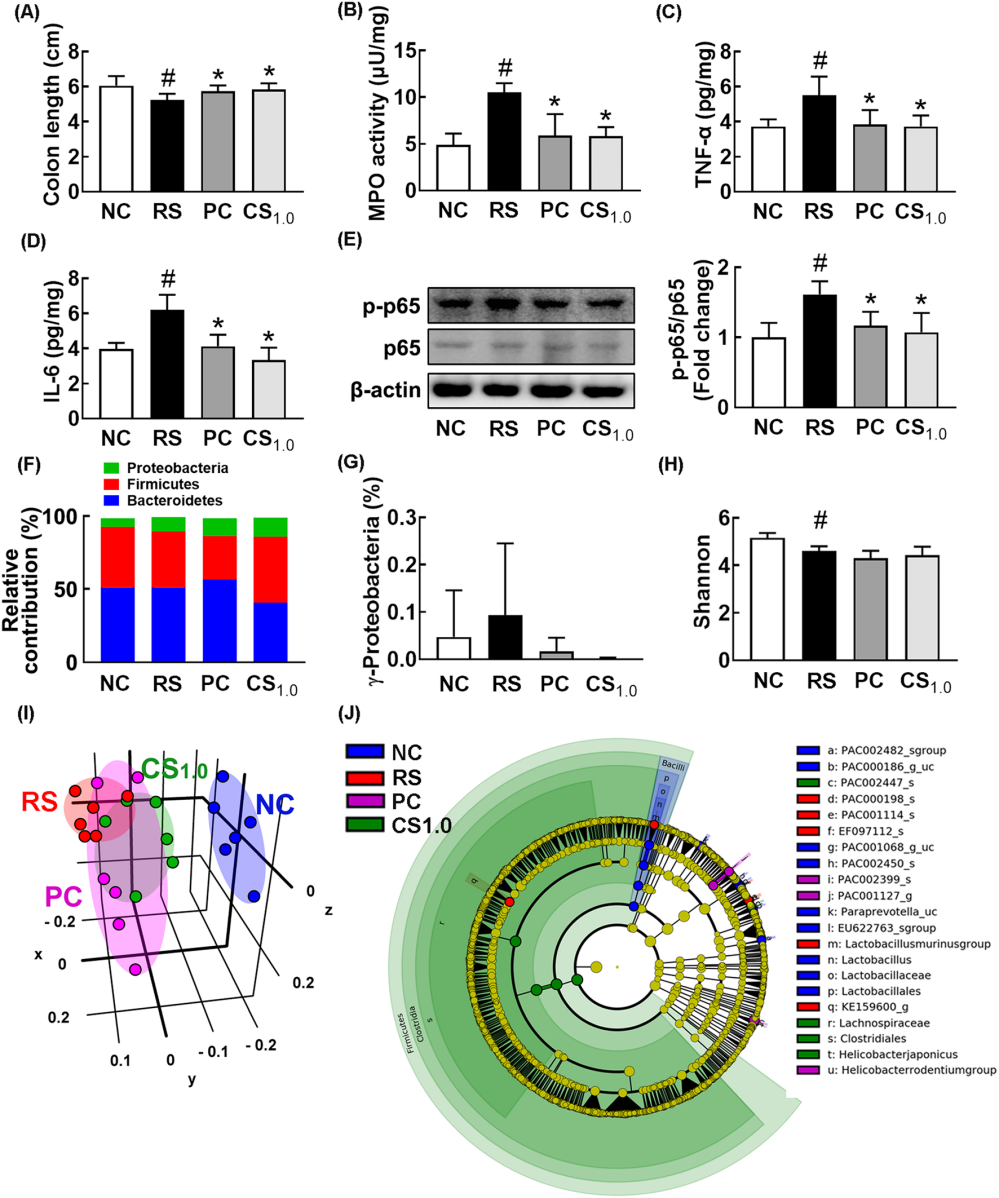
Orally gavage of CSS alleviated restraint stress (RS)-induced colitis and gut dysbiosis in mice. Effects on RS-induced colon shortening (**A**), myeloperoxidase (MPO) activity (**B**), TNF-α (C), and IL-6 expression (**D**), and NF-κB activation (p-p65 to p65 ratio) (**E**) in the colon. Effects on the composition of gut microbiota: phylum (**F**), γ-Proteobacteria level (**G**), α-diversity (Shannon index) (**H**), β-diversity (principal coordinate analysis [PCoA] plot, **H**) based on weighted pairwise Fast UniFrac analysis (**I**), and Cladogram (**J**). Cladogram was generated by LEfSE indicating significant differences in gut microbial abundances among normal control (NC, blue), RS-treated (RS, red), buspirone-treated (PC, purple), and CSS-treated (CS1.0, green) groups. Yellow nodes represent species with no significant difference. The threshold logarithmic score set at 3.5 in the family level and ranked. Test agents (RS, vehicle [saline]; PC, (1 mg/kg/day of buspirone, i.p; CS_1.0_, 1 g/kg/day of CSS) were treated daily for 5 days from the next day after the final exposure to restraint stress (RS). Normal control mice (NC) was orally treated with vehicle (saline) instead of test agents. Data values were indicated as mean ± SD (n = 6). ^#^*p* < 0.05 *vs.* NC group. ^*^p < 0.05 *vs*. RS group

Next, we investigated the anti-colitis effect of CSS in mice with RS-induced anxiety and depression. Exposure to RS led to colon shortening, induced myeloperoxidase activity and NF-κB activation (p-p65 to p65 ratio), and increased TNF-α and IL-6 expression in the colon, leading to the outbreak of colitis (Fig. 3A–E). However, CSS treatment alleviated the RS-induced colitis: it inhibited colon shortening, suppressed myeloperoxidase activity and NF-κB activation (p-p65 to p65 ratio), and decreased IL-6 expression.

To understand whether CSS could alleviate gut dysbiosis, we investigated the effect of CSS on the gut dysbiosis in mice with RS-induced anxiety and depression. Exposure to RS significantly suppressed the bacterial α-diversity, as demonstrated by Shannon’s diversity index, whereas the β-diversity was shifted by using the principal coordinate analysis (PCoA) (Fig. 3H, I). Oral administration of CSS partially shifted RS-shifted β-diversity into those of the control group, while the α-diversity was weakly, but not significantly, affected. Furthermore, exposure to RS significantly altered gut microbiota composition: the fecal microbiota composition of RS-exposed mice was significantly different from that of normal control mice (Fig. 3F–J). Exposure to RS suppressed the populations of Actinobacteria, Deferribacteres, and Verrucomicrobia and increased the population of Proteobacteria, particularly γ-Proteobacteria, at the phylum level (Fig. 3F–J, Additional file 1: Table S1–S3). CSS treatment partially restored RS-suppressed Deferribacteres, Verrucomicrobia, and γ-Proteobacteria population, populations. At the family level, RS exposure suppressed AC160630_f, Lactobacillaceae, and Prevotellaceae populations and increased Desulfovibrionaceae population. However, CSS treatment partially restored RS-suppressed AC160630_f, Prevotellaceae, and Lactobacillaceae population and RS-increased Desulfovibrionaceae population. At the genus level, CSS treatment partially restored RS-induced LT706945_g and PAC001091_g populations and RS-suppressed Lactobacillus and Prevotella populations.

### FMT from RS/CSS-treated mice alleivated anxiety, depression, and colitis in the transplanted mice with RS-induced anxiety and depression

**Fig. 4:**
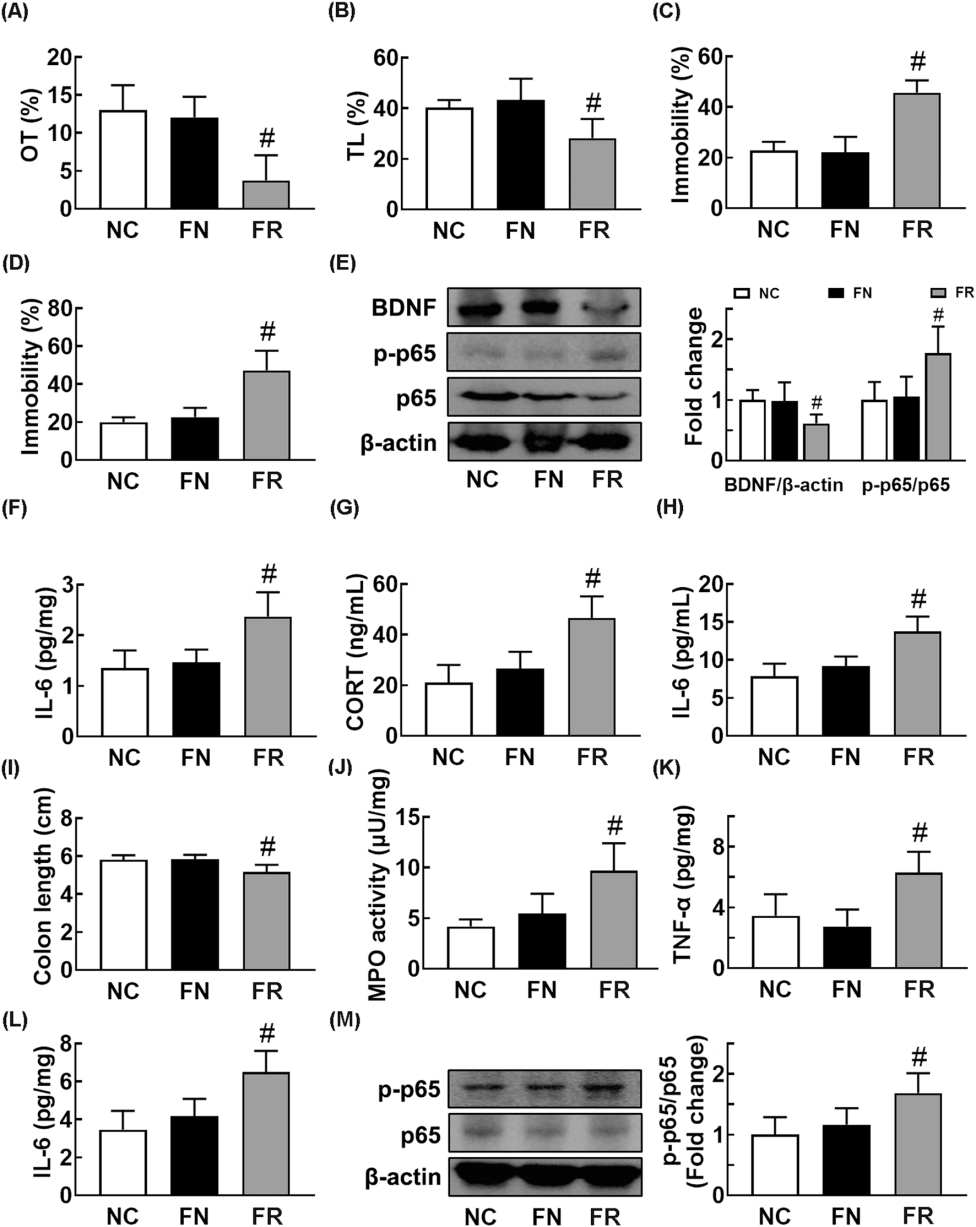
Effects of fecal transplantations of normal control mice or restraint stress (RS)-treated mice on the occurrence of anxiety, depression, and colitis in the transplanted mice. **A** Effects on the time spent in open arm (OT) in the EPM task. **B** Effects on the time spent in the light box (TL) in the LDT task. Effects on the immobility in the TST (**C**) and FST (**D**). Effects on the BDNF expression and NF-κB activation (p-p65 to p65 ratio) (**E**) and IL-6 expression (**F**) in the hippocampus. Effect on corticosterone (CORT, **G**) and IL-6 (H) in the blood. Effects on colon length (**I**), myeloperoxidase activity (**J**), TNF-α and IL-6 expression, and NF-κB activation in the colon. FN and FR groups were treated with the fecal microbiota of normal mice and RS-treated mice daily for 5 days, respectively. NC group was orally treated with vehicle (saline) instead of the fecal microbiota. Data values were indicated as mean ± SD (n = 6). ^#^p < 0.05 vs. NC group

Next*,* to understand the effect of the FMT on the outbreak of anxiety, depression, and colitis, we transplanted the feces of normal control mice (FN) or RS-exposed mice (FR) in control mice and monitored anxeity- and depression-like behaviors (Fig. 4A–D). FR transplantation significanlty increased anxiety- and depression-like behaviors, IL-6 expression in the hippocampsus, IL-6 and corticosterone levels in the blood, TNF-α and IL-6 expression, myeloperoxidase activity, and NF-κB activation (p-p65 to p65 ratio) in the colon, while the FN transplantation did not significantly affect them (Fig. 4E–M).

**Fig. 5:**
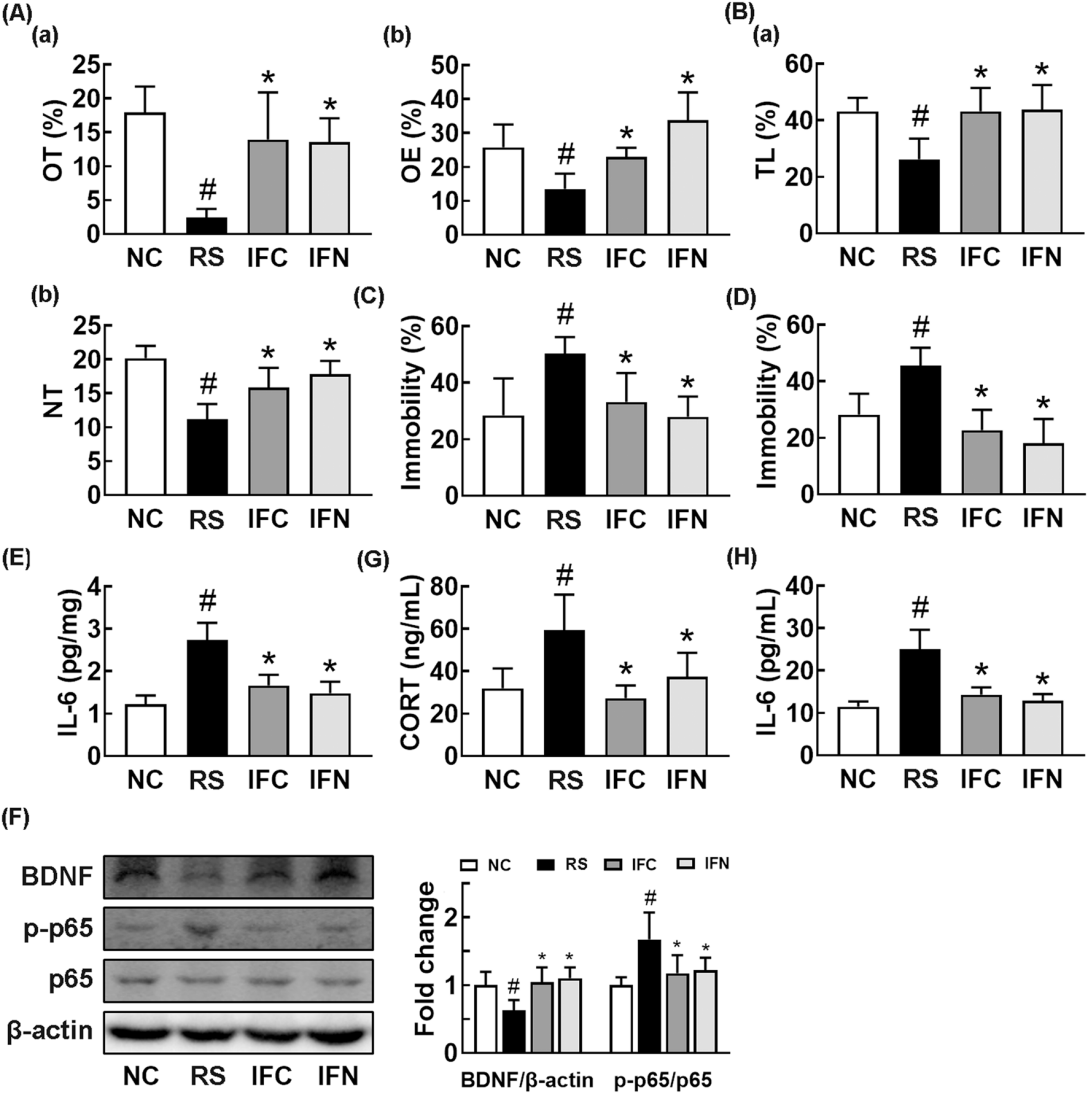
Fecal transplantations of normal control and CSS/RS-treated mice alleviated restraint stress (RS)-induced anxiety and depression in the transplanted mice. Effects on RS-induced anxiety/depression-like behaviors in the EPM task (**A**:** a**, time spent in open arm [OT];** b**, open arm entries [OE]), LDT (**B**; **a**, time spent in the light box [TL]; **b**, number of transition into the light box entry [NT]), TST (**C**), and FST (**D**). Effects on IL-6 (E) and NF-κB activation (p-p65 to p65 ratio) (**F**) in the hippocampus. Effect on corticosterone (**G**) and IL-6 (**H**) in the blood. RS, IFC, and IFN groups were treated with vehicle (saline), fecal microbiota of CSS/RS-treated mice (FC), and fecal microbiota of normal control mice (FN) in mice with RS-induced depression daily for 5 days from 24 h after the exposure to RS, respectively. NC group was orally treated with vehicle (saline) in normal control mice instead of the fecal sample. Data values were indicated as mean ± SD (n = 6). ^#^p < 0.05 vs. Con group. *p < 0.05 vs. RS group

**Fig. 6:**
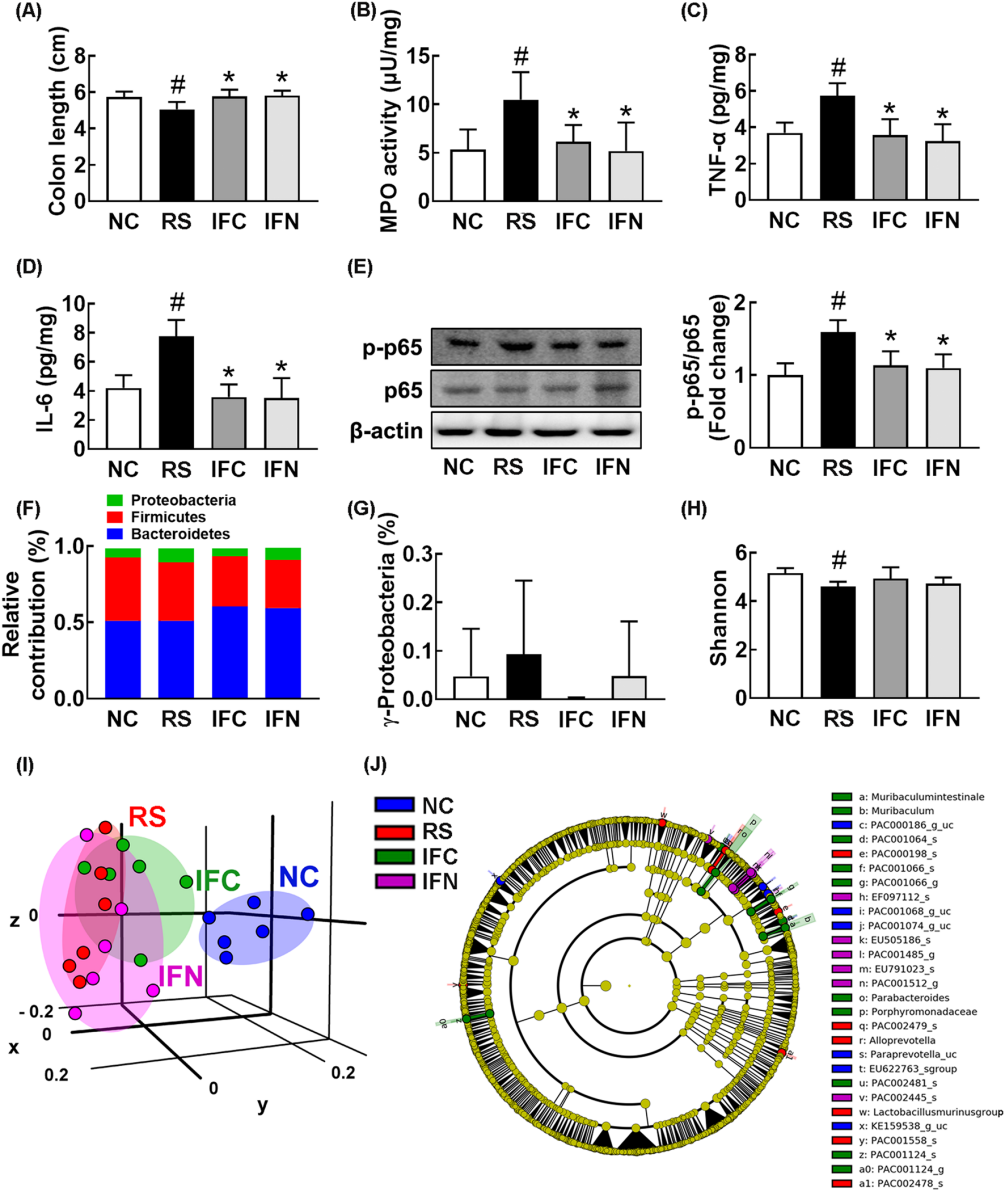
Fecal transplantations of normal control and CSS/RS-treated mice alleviated restraint stress (RS)-induced colitis and gut dysbiosis in the transplanted mice. Effects on RS-induced colon shortening (**A**), myeloperoxidase (MPO) activity (**B**), TNF-α (**C**), and IL-6 expression (**D**), and NF-κB activation (p-p65 to p65 ratio) (**E**) in the colon. Effects on the composition of gut microbiota: phylum (**F**), γ-Proteobacteria level (**G**), α-diversity (Shannon index) (**H**), β-diversity (principal coordinate analysis [PCoA] plot, H) based on weighted pairwise Fast UniFrac analysis (**I**), and Cladogram (**J**). Cladogram was generated by LEfSE indicating significant differences in gut microbial abundances among normal control (NC, blue), RS-treated (RS, red), CSS/RS-treated mouse feces-transplanted (IFC, purple), and normal control mouse feces-transplanted (IFN) groups. Yellow nodes represent species with no significant difference. The threshold logarithmic score set at 3.5 in the family level and ranked. RS, IFC, and IFN groups were treated with vehicle (saline), fecal microbiota of CSS/RS-treated mice (FC), and fecal microbiota of normal control mice (FN) in mice with RS-induced depression daily for 5 days from 24 h after the exposure to RS, respectively. NC group was orally treated with vehicle (saline) in normal control mice instead of the fecal sample. Data values were indicated as mean ± SD (n = 6). ^#^*p* < 0.05 *vs.* NC group. ^*^p < 0.05 *vs*. RS group

Therefore, to interpret gut microbiota-involved anti-depressive effect of CSS, we tranplanted the fecal microbiota of normal control (FN) and RS/CSS-treated mice (FC) in mice with RS-induced anxiety and depression and investigated their effects on the depression, anxiety, and colitis (Fig. 5). The FN transplantation into mice with RS-induced anxiety and depression markedly inhibited the RS-induced anxity- and depression-like behaviors, NF-κB activation in the hippocampus, and IL-6 and corticosterone levels in the blood (Fig. 5A–F). The FC transplantation also markedly inhibited the RS-induced anxity- and depression-like behaviors, suppressed NF-κB activation (p-p65 to p65 ratio) in the hippocampus, and reduced IL-6 and corticosterone levels in the blood (Fig. 5G,H). These FMTs inhibited the RS-induced colitis: they decreased myeloperoxidase activity and IL-6 expression and suppressed NF-κB activation (Fig. 6A–E).

Furthermore, we investigated the effects of FN and FC on the gut microbiota composition in RS-exposed mice (Fig. 6F–J, Additional file 1: Tables S4–S6). Transplantation of FN or FC partially modulated RS-shifted β-diversity into those of the control group, whereas the α-diversity was weakly, but not significantly, affected. The fecal bacterial composition between mice with and without exposure to RS showed the significant difference. At the phylum, treatment with FC or FN suppressed RS-induced population of Proteobacteria, particularly γ-Proteobacteria, and increased RS-suppressed population of Verrucomicrobia. At the family level, treatment with FC or FN increased RS-suppressed Lactobacillaceae and Prevotellaceae population and suppressed RS-induced Desulfovibrionaceae population. At the genus level, their treatments increased RS-suppressed Lactobacillus and Paraprevotella populations and reduced RS-induced PAC001091_g population.

## Discussion

Gut microbiota are closely connected with the function and development of brain [26, 27]. Recent studies have focused the physiological function of gut microbiota on the occurrence of psychiatric disorders through the microbiota-gut-brain (MGB) axis [26, 27]. The induction of gut inflammation by 2,4,6-trinitrobenzenesulfonic acid, the representative colitis inducer, occurs memory impairment in mice [28]. The induction of anxiety and depression by exposure to stressors such as IS and antibiotic ampicillin occurs gut inflammation and dysbiosis in mice [11, 23]. The fecal transplantation of stressors-induced mice also occurs gut inflammation and dysbiosis and psychiatric disorder in the transplanted mice [11]. However, the amelioration of gut dysbiosis by probiotics and attenuation of gut inflammation by natural products improve psychiatric disorders in mice [28, 29].

In the present study, exposure to RS raised neuroinflammation and colitis: it induced IL-6 expression and NF-κB activation in the hippocampus and myeloperoxidase activity and NF-κB activation in the colon. It also increased IL-6 and corticosterone levels in the blood, which is positively correlated with stress index score [30]. RS exposure caused anxiety/depression. Moreover, RS exposure caused gut dysbiosis: it significantly suppressed the bacterial α-diversity and shifted the β-diversity. In particular, RS increased the γ-Proteobacteria population. Gut bacteria belonging to γ-Proteobacteria such as *Escherichia coli* and *Klebsiella oxytoca* cause anxiety, depression, and colitis in mice by the excessive production of bacterial LPS in the gut [11, 21]. However, Lactobacilli alleviated stressor-induced depression and colitis in mice [11, 21]. Bailey et al. reported that exposure to stressors decreased the populations of Bacteroides sp and Clostridium sp. in the gut, induced the expression of inflammatory cytokines, and increased neuroendocrine hormone levels in the brain of conventional mice [29]. The overgrowth of Proteobacteria, particularly γ-Proteobacteria, including *Escherichia coli* population by exposure to 2,4,6-trinitrobenzenesulfonic acid raised the colitis and gut dysbiosis in mice, leading to the outbreak of cognitive impairment [28]. Kim et al. reported that oral treatment with *Escherichia coli* caused anxiety, depression, colitis, and gut dysbiosis in mice [25]. These results suggest that exposure to stressors such as RS can cause colitis with anxiety and depression by the induction of gut dysbiosis.

We found that CSS alleviated RS-induced anxiety- and depression-like behaviors, as reported previously [16, 17]. CSS at a dose of 1.0 mg/kg showed the most anti-depressive effect. The high dose attenuated its effect. This results suggest that the dose may be show the toxic effects such as excessive sedative. CSS also increased the stressor-suppressed expression of BDNF in the hippocampus, reduced the stressor-induced NF-κB activation and TNF-α and IL-6 expression in the hippocampus and colon, and decreased IL-6, and corticosterone levels in the blood. Li et al. reported that CSS alleviated depressive behaviors and decreased adrenocorticotropic and corticotropin-releasing hormones in chronic stress-exposed rats [18]. Deng et al. reported that CSS increased the expression of TrkB and BDNF in the amygdala and hippocampus of chronic stress-exposed rats [31]. Xu et al. reported that BDNF is regulated via MyD88/NF-κB signaling [32]. These results suggest that CSS may mitigate anxiety and depression by increasing NF-κB-mediated BDNF expression.

In the present study, CSS also alleviated RS-induced gut dysbiosis with colitis in mice. In particular, CSS treatment increased RS-suppressed Lactobacillaceae population in the gut microbiota while the RS-induced Proteobacteria population was not affected. Exposure to high-fat diet or RS induces colitis and gut Proteobacteria population in mice, while the Lactobacilli population was reduced [11, 33]. However, oral gavage of commensal *Lactobacillus johnsonii* suppressed Proteobacteria- or RS-induced anxiety and colitis in mice [11]. Liang et al. reported that CSS suppressed the Enterobacteriaceae population in the feces of rat with high fat diet-induced obesity [19]. However, Yu et al. reported that chronic exposure to imipenem/cilastatin increased Proteobacteria and Bacteroidetes populations and suppressed the Firmicutes in the gut microbiota of rats [20]. They also reported that oral gavage of CSS alleviated stress/antibiotics-induced depression in rats by suppressing stress/antibiotics-induced Oscillibacter population and increasing stress/antibiotics-suppressed Bacteroidetes population. These results suggest that CSS can mitigate depression by modulating gut microbiota. Jang et al. reported that the induction of Proteobacteria, particularly Enterobacteriaceae, by antibiotic ampicillin significantly caused anxiety/depression in mice and the suppression of Proteobacteria by Lactobacilli alleviated anxiety/depression [21]. However, Yu et al. reported that CSS increased stress/antibiotics-suppressed Firmicutes and Proteobacteria populations in mice, resulting in the amelioration of depression [20]. To clarify the discrepancy, we examined the fecal transplantation of FN, FR, and FC in mice with RS-induced depression.

The FR transplantation caused anxiety/depression with colitis in the transplanted mice, as previously reported [11]. They also reported that FR transplantation significantly induced the Proteobacteria population in the gut microbiota. We also found that oral gavage of CSS or transplantation of FC or FN suppressed gut γ-Proteobacteria population and increased gut Lactobacillaceae population in mice with RS-induced depression. Of these treatments, CSS treatment and CSS/RS-treated mouse feces, FC, transplantation more potently suppressed gut γ-Proteobacteria population than FN, normal mouse feces, transplantation and buspirone treatment. Transplantation of CSS/RS-treated mouse feces, which were collected 2 days after the final CSS treatment in RS-exposed mice to prepare CSS-free feces, and oral gavage of CSS suppressed RS-induced anxiety, depression, and colitis in the transplanted mice: they significantly alleviated anxiety- and depression-like behaviors, suppressed NF-κB activation and BDNF and IL-6 expression in the hippocampus. Its treatment also reduced RS-induced IL-6 and corticosterone levels in the blood and IL-6 and suppressed NF-κB activation and myeloperoxidase activity in the colon. These results suggest that CSS may mitigate anxiety, depression, and colitis by regulating gut microbiota. In particular, CSS suppressed the ratio of inflammatory γ-Proteobacteria to antiinflammatory Lactobacillaceae can decrease the expression of proinflammatory cytokines in the gut through the suppression of NF-κB activation, resulting in the alleviation of depression through the attenuation of systemic inflammation including neuroinflammation.

Exposure to stressors more exaggeratedly causes anxiety-like behaviors in germ-free mice than in conventional ones [34, 35]. The FMT from conventional mice into germ-free mice attenuates the hyperactive anxiety in transplanted mice [36]. Exposure to antibiotic ampicillin also occurs anxiety/depression in mice [21]. The transplantation of beneficial gut bacteria into mice with ampicillin-induced depression alleviates anxiety in transplanted mice [21]. However, gut bacteria *Klebsiella oxytoca* and *Escherichia coli* cause anxiety with the suppression of BDNF expression in SPF mice [11, 21]. The gut dysbiosis suppresses the expression of BDNF in the hippocampus of mice [37]. Moreover, the FMT of FC or FN suppressed NF-κB activation and IL-6 expression in the hippocampus and colon, while the BDNF expression was increased. These results suggest that gut dysbiosis can cause neuroinflammation and colitis by inducing the proinflammatory cytokine expression through NF-κB activation, resulting in the occurrence of depression and CSS can suppress NF-kB activation-induced gut and systemic inflammation through the alleviation of gut dysbiosis, particularly the reduction of inflammatory γ-Proteobacteria to antiinflammatory Lactobacillaceae population ratio, resulting in the alleviation of anxiety and depression by inducing BDNF expression through the suppression of IL-6 expression.

## Conclusions

CSS significantly reduced the RS-induced anxiety- and depression-like behaviors and hippocampal NF-κB activation and IL-6 expression, blood corticosterone level, and colonic IL-6 expression and myeloperoxidase activity, while the RS-suppressed BDNF expression increased. CSS decreased the RS-induced γ-Proteobacteria population in gut microbiota, while the RS-suppressed Lactobacillaceae, Prevotellaceae, and AC160630_f populations increased. Fecal transplantation of normal control or RS/CSS-treated mice into RS-exposed mice significantly mitigated RS-induced anxiety/depression, colitis, and gut dysbiosis. CSS may alleviate anxiety and depression by regulating gut microbiota and NF-κB-involved BDNF expression.

## Supplementary Information

**Additional file 1: Table S1.** Effects of Chaihushugansan (CS1.0) and buspirone (PC) on the gut microbiota composition at the phylum level in mice with RS-induced anxiety/depression. **Table S2.** Effects of Chaihushugansan (CS1.0) and buspirone (PC) on the gut microbiota composition at the family level in mice with RS-induced anxiety/depression. **Table S3.** Effects of Chaihushugansan (CS1.0) and buspirone (PC) on the gut microbiota composition at the genus level in mice with RS-induced anxiety/depression. **Table S4.** Effects of Chaihushugansan (CS1.0)-treated mouse feces and normal control mouse feces transplantations on the gut microbiota composition at the phylum level in mice with RS-induced anxiety/depression. **Table S5.** Effects of Chaihushugansan (CS1.0)-treated mouse feces and normal control mouse feces transplantations on the gut microbiota composition at the family level in mice with RS-induced anxiety/depression. **Table S6.** Effects of Chaihushugansan (CS1.0)-treated mouse feces and normal control mouse feces transplantations on the gut microbiota composition at the genus level in mice with RS-induced anxiety/depression.

## Data Availability

Pyrosequencing reads were deposited in the short read archive of NCBI under accession number PRJNA649086.

## Abbreviations

BDNF: Brain-derived neurotrophic factor
CNS: Central nervous systems
CSS: Chaihu-Shugan-San
ELISA: Enzyme-linked immunosorbent assay
EPM: Elevated plus maze
FC: Restraint stress/Chaihu-Shugan-San-treated mouse feces
FR: Restraint stress-treated mouse feces
FN: Normal control mouse feces
FST: Forced swimming test
HPA: Hypothalamo-pituitary-adrenal
IL: Interleukin
LDT: Light/dark transition
LT: Time spent in the light box
MGB: Microbiota-gut-brain
NC: Normal control
OT: Time spent in open arm
RIPA: Radioimmunoprecipitation assay
RS: Restraint stress
TNF: Tumor necrosis factor
TST: Tail suspension test
