# Supplementary material for: Chaihu-Shugan-San (Shihosogansan) alleviates restraint stress-generated anxiety and depression in mice by regulating NF-κB-mediated BDNF expression through the modulation of gut microbiota
Source: Chin Med. 2021 Aug 14;16:77. doi: 10.1186/s13020-021-00492-5 (PMC8364688; doi:10.1186/s13020-021-00492-5)
Supplement: Supplementary file 1 — Additional file 1: Table S1. Effects of Chaihushugansan (CS1.0) and buspirone (PC) on the gut microbiota composition at the phylum level in mice with RS-induced anxiety/depression. Table S2. Effects of Chaihushugansan (CS1.0) and buspirone (PC) on the gut microbiota composition at the family level in mice with RS-induced anxiety/depression. Table S3. Effects of Chaihushugansan (CS1.0) and buspirone (PC) on the gut microbiota composition at the genus level in mice with RS-induced anxiety/depression. Table S4. Effects of Chaihushugansan (CS1.0)-treated mouse feces and normal control mouse feces transplantations on the gut microbiota composition at the phylum level in mice with RS-induced anxiety/depression. Table S5. Effects of Chaihushugansan (CS1.0)-treated mouse feces and normal control mouse feces transplantations on the gut microbiota composition at the family level in mice with RS-induced anxiety/depression. Table S6. Effects of Chaihushugansan (CS1.0)-treated mouse feces and normal control mouse feces transplantations on the gut microbiota composition at the genus level in mice with RS-induced anxiety/depression. [file 13020_2021_492_MOESM1_ESM.docx]

**[Additional information]**

**Chaihu-Shugan-San (Shihosogansan) alleviates restraint stress-generated anxiety and depression in mice by regulating NF-κB-mediated BDNF expression through the modulation of gut microbiota**

Sang-Kap Han, Jeon-Kyung Kim, Hee-Seo Park, Yeun-Jeong Shin, and Dong-Hyun Kim^*^

*Neurobiota Research Center and Department of Life and Nanopharmaceutical Sciences, College of Pharmacy, Kyung Hee University, 26, Kyungheedae-ro, Dongdaemun-gu, Seoul 02447, Korea*

Table S1. Effects of Chaihushugansan (CS1.0) and buspirone (PC) on the gut microbiota composition at the phylum level in mice with IS-induced anxiety/depression

| Taxon Name | Composition (%)^a^ | | | |
| --- | --- | --- | --- | --- |
|  | NC^b^ | RS | PC | CS1.0 |
| Actinobacteria | 0.23 ± 0.08 | 0.01 ± 0.01^#^ | 0.01 ± 0.01 | 0.00 ± 0.01 |
| Bacteroidetes | 51.06 ± 10.92 | 50.98 ± 7.26 | 56.36 ± 21.61 | 40.51 ± 14.22 |
| Deferribacteres | 0.55 ± 0.78 | 0.13 ± 0.13 | 0.22 ± 0.12 | 0.79 ± 0.90 |
| Firmicutes | 41.54 ± 11.05 | 38.17 ± 5.78 | 29.90 ± 9.47 | 45.10 ± 9.08 |
| Proteobacteria | 5.86 ± 3.59 | 10.01 ± 3.80 | 12.00 ± 14.91 | 13.10 ± 8.35 |
| Verrucomicrobia | 0.23 ± 0.22 | 0.05 ± 0.06 | 0.95 ± 2.27 | 0.10 ± 0.14 |

^a^Mean ± SD. ^b^NC, normal control; RS, restraint stress-exposed group; PC, buspirone-treated mice with RS; CS1.0, RS mice treated with 1 g/kg of CSS. ^#^*p*<0.05 vs. NC group **p*<0.05 vs. IS group

Table S2. Effects of Chaihushugansan (CS1.0) and buspirone (PC) on the gut microbiota composition at the family level in mice with IS-induced anxiety/depression

| Taxon Name | Composition (%)^a^ | | | |
| --- | --- | --- | --- | --- |
|  | NC^b^ | RS | PC | CS1.0 |
| AC160630_f | 1.21 ± 0.67 | 0.36 ± 0.41^#^ | 0.58 ± 0.40 | 0.41 ± 0.34 |
| Bacteroidaceae | 7.09 ± 2.75 | 5.66 ± 2.77 | 7.04 ± 6.07 | 5.44 ± 2.60 |
| Desulfovibrionaceae | 1.11 ± 0.50 | 3.04 ± 1.52^#^ | 1.53 ± 0.95 | 1.94 ± 1.10 |
| Helicobacteraceae | 4.23 ± 2.80 | 6.60 ± 3.55 | 10.21 ± 15.17 | 10.76 ± 8.35 |
| Lachnospiraceae | 27.92 ± 11.94 | 26.87 ± 7.76 | 20.43 ± 6.44 | 33.17 ± 13.07 |
| Lactobacillaceae | 2.69 ± 1.52 | 0.18 ± 0.11^#^ | 0.23 ± 0.14 | 0.56 ± 0.61 |
| Muribaculaceae | 28.01 ± 6.61 | 32.54 ± 5.19 | 32.14 ± 11.52 | 23.07 ± 9.73 |
| Prevotellaceae | 9.81 ± 5.40 | 5.94 ± 2.91 | 10.42 ± 8.09 | 8.04 ± 6.00 |
| Rikenellaceae | 4.00 ± 1.83 | 5.17 ± 3.61 | 4.91 ± 4.25 | 2.66 ± 0.86 |
| Ruminococcaceae | 9.97 ± 1.26 | 9.81 ± 2.33 | 8.02 ±3.20 | 10.25 ± 5.32 |

^a^Mean ± SD. ^b^NC, normal control; RS, restraint stress-exposed group; PC, buspirone-treated mice with RS; CS1.0, RS mice treated with 1 g/kg of CSS. ^#^*p*<0.05 vs. NC group **p*<0.05 vs. IS group

Table S3. Effects of Chaihushugansan (CS1.0) and buspirone (PC) on the gut microbiota composition at the genus level in mice with IS-induced anxiety/depression

| Taxon Name | Composition (%)^a^ | | | |
| --- | --- | --- | --- | --- |
|  | NC^b^ | RS | PC | CS1.0 |
| Alistipes | 3.55 ± 1.79 | 4.55 ± 3.04 | 4.36 ± 3.82 | 2.28 ± 0.67 |
| Bacteroides | 7.09 ± 2.75 | 5.65 ± 2.77 | 7.02 ± 6.03 | 5.44 ± 2.60 |
| Helicobacter | 4.23 ± 2.79 | 6.59 ± 3.55 | 10.18 ± 15.09 | 10.75 ± 8.34 |
| KE159538_g | 10.90 ± 10.82 | 5.20 ± 3.96 | 4.03 ± 3.52 | 15.38 ± 17.15 |
| LT706945_g | 0.73 ± 0.60 | 2.70 ± 1.56^#^ | 1.33 ± 0.93 | 1.64 ± 1.09 |
| Lactobacillus | 2.68 ±1.52 | 0.18 ± 0.11^#^ | 0.23 ± 0.14 | 0.56 ± 0.61 |
| Oscillibacter | 2.68 ± 1.16 | 2.72 ± 1.16 | 2.47 ± 1.84 | 2.47 ± 1.34 |
| PAC000186_g | 9.23 ± 3.73 | 9.31 ± 2.01 | 6.33 ± 2.91 | 6.41 ± 3.79 |
| PAC000198_g | 1.83 ± 0.55 | 3.77 ± 2.59 | 3.19 ± 1.72 | 1.15 ± 0.60* |
| PAC000664_g | 2.39 ± 1.19 | 3.03 ± 2.20 | 2.60 ± 1.18 | 2.08 ± 0.91 |
| PAC001068_g | 6.46 ± 2.32 | 9.35 ± 2.11^#^ | 11.79 ± 7.26 | 8.19 ± 3.12 |
| PAC001091_g | 0.63 ± 0.56 | 5.52 ± 3.23^#^ | 2.74 ± 2.99 | 2.24 ± 3.07 |
| Paraprevotella | 4.23 ± 3.79 | 0.99 ± 0.79 | 7.58 ± 7.02* | 2.28 ± 2.05 |
| Prevotella | 2.54 ± 2.12 | 1.72 ± 1.59 | 1.59 ± 1.01 | 3.17 ± 4.29 |
| Pseudoflavonifractor | 2.93 ± 1.20 | 2.47 ± 0.73 | 2.70 ± 1.00 | 2.29 ± 0.64 |

^a^Mean ± SD. ^b^NC, normal control; RS, restraint stress-exposed group; PC, buspirone-treated mice with RS; CS1.0, RS mice treated with 1 g/kg of CSS. ^#^*p*<0.05 vs. NC group **p*<0.05 vs. IS group

Table S4. Effects of Chaihushugansan (CS1.0)-treated mouse feces and normal control mouse feces transplantations on the gut microbiota composition at the phylum level in mice with IS-induced anxiety/depression

| Taxon Name | AVE ± SD | | | |
| --- | --- | --- | --- | --- |
|  | NC | RIS | IFC | IFN |
| Actinobacteria | 0.23 ± 0.08 | 0.01 ± 0.01^#^ | 0.01 ± 0.01 | 0.01 ± 0.01 |
| Bacteroidetes | 51.06 ± 10.92 | 50.98 ± 7.26 | 60.24 ± 15.48 | 59.29 ± 12.96 |
| Deferribacteres | 0.55 ± 0.78 | 0.13 ± 0.13 | 0.03 ± 0.06 | 0.05 ± 0.05 |
| Firmicutes | 41.54 ± 0.05 | 38.17 ± 5.78 | 33.16 ± 12.92 | 31.48 ± 7.91 |
| Proteobacteria | 5.86 ± 7.91 | 10.01 ± 3.80 | 5.19 ± 3.30* | 8.00 ± 5.53 |
| Verrucomicrobia | 0.23 ± 0.22 | 0.05 ± 0.06 | 0.45 ± 0.64 | 0.10 ± 0.16 |

^a^Mean ± SD. ^b^NC, normal control; RIS, mice transplanted with the feces of RS mice; IFC, mice transplanted with the feces of CSS/RS-treated mice; IFN, mice transplanted with the feces normal control mice. ^#^*p*<0.05 vs. NC group **p*<0.05 vs. IS group

Table S5. Effects of Chaihushugansan (CS1.0)-treated mouse feces and normal control mouse feces transplantations on the gut microbiota composition at the family level in mice with IS-induced anxiety/depression

| Taxon Name | AVE ± SD | | | |
| --- | --- | --- | --- | --- |
|  | NC | RIS | IFC | IFN |
| Bacteroidaceae | 7.09 ± 2.75 | 5.66 ± 2.77 | 5.76 ± 3.10 | 3.07 ± 2.41 |
| Christensenellaceae | 0.27 ± 0.23 | 0.75 ± 0.37^#^ | 0.26 ± 0.11* | 1.31 ± 1.77 |
| Desulfovibrionaceae | 1.11 ± 0.50 | 3.04 ± 1.52^#^ | 1.28 ± 0.73* | 1.40 ± 0.82* |
| Helicobacteraceae | 4.23 ± 2.80 | 6.60 ± 3.55 | 3.21 ± 3.31 | 6.29 ± 4.73 |
| Lachnospiraceae | 27.92 ± 11.94 | 26.87 ± 7.76 | 20.27 ± 11.65 | 19.63 ± 7.39 |
| Lactobacillaceae | 2.69 ± 1.52 | 0.18 ± 0.11^#^ | 0.98 ± 1.17 | 0.47 ± 0.46 |
| Muribaculaceae | 28.01 ± 6.61 | 32.54 ± 5.19 | 34.35 ± 8.67 | 37.38 ± 13.73 |
| Prevotellaceae | 9.81 ± 5.40 | 5.94 ± 2.91 | 15.05 ± 7.61* | 9.42 ± 7.12 |
| Rikenellaceae | 4.00 ± 1.83 | 5.17 ± 3.61 | 3.23 ± 1.62 | 6.67 ± 5.13 |
| Ruminococcaceae | 9.97 ± 1.26 | 9.81 ± 2.33 | 11.24 ± 4.06 | 9.38 ± 2.61 |

^a^Mean ± SD. ^b^NC, normal control; RIS, mice transplanted with the feces of RS mice; IFC, mice transplanted with the feces of CSS/RS-treated mice; IFN, mice transplanted with the feces normal control mice. ^#^*p*<0.05 vs. NC group **p*<0.05 vs. IS group

Table S6. Effects of Chaihushugansan (CS1.0)-treated mouse feces and normal control mouse feces transplantations on the gut microbiota composition at the genus level in mice with IS-induced anxiety/depression

| Taxon Name | Composition (%)^a^ | | | |
| --- | --- | --- | --- | --- |
|  | NC^b^ | RS | IFC | IFN |
| Alistipes | 3.55 ± 1.79 | 4.55 ± 3.04 | 3.03 ± 1.56 | 6.19 ± 4.99 |
| Bacteroides | 7.09 ± 2.75 | 5.65 ± 2.77 | 5.76 ± 3.10 | 3.07 ± 2.40 |
| Helicobacter | 4.23 ± 2.79 | 6.59 ± 3.55 | 3.20 ± 3.30 | 6.28 ± 4.72 |
| KE159538_g | 10.90 ± 10.82 | 5.20 ± 3.96 | 2.29 ± 1.64 | 4.10 ± 3.82 |
| Lactobacillus | 2.68 ± 1.52 | 0.18 ± 0.11^#^ | 0.98 ± 1.17 | 0.47 ± 0.46 |
| Oscillibacter | 2.68 ± 1.16 | 2.72 ± 0.72 | 1.94 ± 1.79 | 2.90 ± 1.90 |
| PAC000186_g | 9.23 ± 3.73 | 9.31 ± 2.01 | 11.32 ± 2.86 | 7.43 ± 2.88 |
| PAC000198_g | 1.83 ± 0.55 | 3.77 ± 2.59 | 2.31 ± 1.35 | 1.25 ± 0.43* |
| PAC000664_g | 2.39 ± 1.19 | 3.03 ± 2.20 | 1.89 ± 1.48 | 1.83 ± 1.18 |
| PAC001068_g | 6.46 ± 2.32 | 9.35 ± 2.11^#^ | 9.78 ± 2.03 | 12.04 ± 5.28 |
| PAC001091_g | 0.63 ± 0.56 | 5.52 ± 3.23^#^ | 1.90 ± 2.07* | 3.07 ± 3.25 |
| Paraprevotella | 4.23 ± 3.79 | 0.99 ± 3.79 | 1.94 ± 1.87 | 1.35 ± 1.42 |
| Prevotella | 2.54 ± 2.12 | 1.72 ± 1.59 | 1.66 ± 1.00 | 1.68 ± 0.96 |
| Pseudoflavonifractor | 2.93 ± 1.20 | 2.47 ± 0.73 | 1.74 ± 0.96 | 2.36 ± 1.36 |
| Ruminococcus | 1.68 ± 1.38 | 1.14 ± 1.56 | 4.02 ± 3.81 | 1.38 ± 1.76 |

^a^Mean ± SD. ^b^NC, normal control; RIS, mice transplanted with the feces of RS mice; IFC, mice transplanted with the feces of CSS/RS-treated mice; IFN, mice transplanted with the feces normal control mice. ^#^*p*<0.05 vs. NC group **p*<0.05 vs. IS group


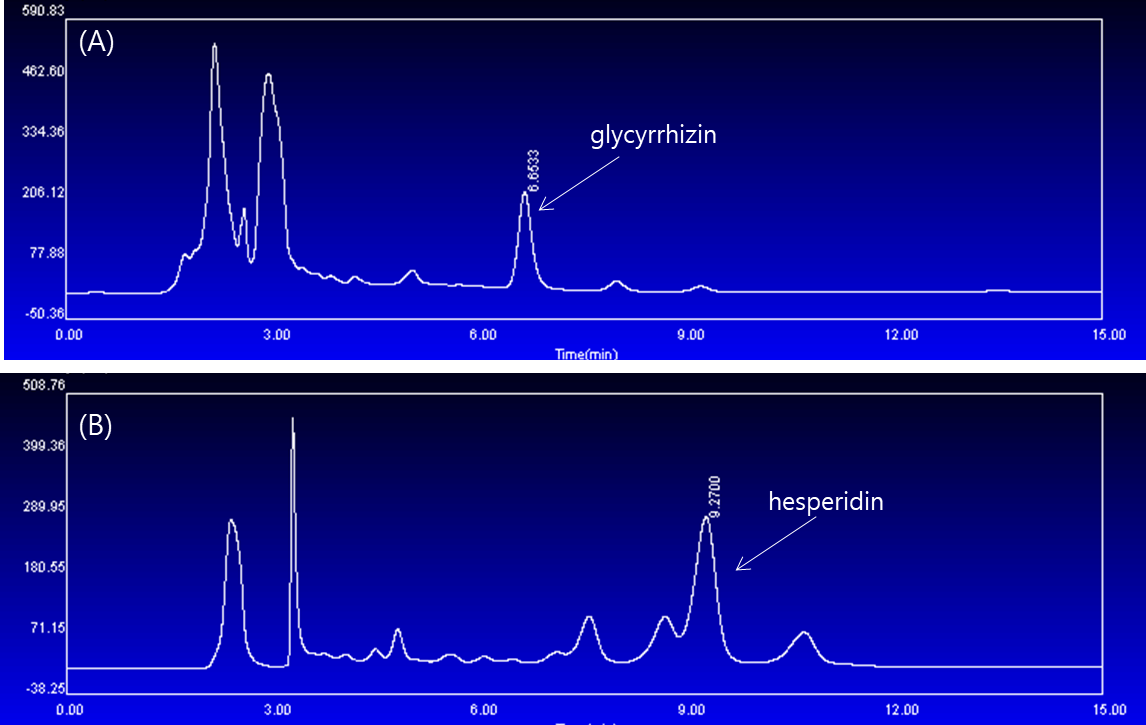


Figure S1. HPLC chromatogram of Chaihushugansan extract. The extract 50 mL was dissolved in 50% ethanol, sonicated, filtrated, and injected in HPLC. (A) HPLC condition: column, Nucelosil C18; elution solvent, DW-acetonitrile-acetic acid (620:380:5); sample injection volume, 10 μL; flow rate, 1. 2mL/min; detection wavelength, 254 nm. (B) HPLC condition: column, Nucelosil C18; elution solvent, 1/15 KH_2_PO_4_: acetonitrile (815:185); sample injection volume, 10 μL; flow rate, 1. 2mL/min; detection wavelength, 280 nm.
